# Drug repurposing: re-inventing therapies for cancer without re-entering the development pipeline—a review

**DOI:** 10.1186/s43046-022-00137-0

**Published:** 2022-08-08

**Authors:** Shafina Siddiqui, Ankita Jaywant Deshmukh, Priyanka Mudaliar, Apoorva Jagannath Nalawade, Deepak Iyer, Jyotirmoi Aich

**Affiliations:** 1 School of Biotechnology and Bioinformatics, DY Patil Deemed to Be University, CBD Belapur, Navi Mumbai, Maharashtra 400 614 India; 2 Laboratory Medicine Program, Toronto General Hospital, University Health Network, University of Toronto, Toronto, Canada

**Keywords:** Cancer, Drug resistance, Drug repurposing, Anti-psychotic, Anti-inflammatory, Anti-viral, Anti-diabetic, Anti-bacterial, Anti-viral

## Abstract

While majority of the current treatment approaches for cancer remain expensive and are associated with several side effects, development of new treatment modalities takes a significant period of research, time, and expenditure. An alternative novel approach is drug repurposing that focuses on finding new applications for the previously clinically approved drugs. The process of drug repurposing has also been facilitated by current advances in the field of proteomics, genomics, and information computational biology. This approach not only provides cheaper, effective, and potentially safer drugs with less side effects but also increases the processing pace of drug development. In this review, we wish to highlight some recent developments in the area of drug repurposing in cancer with a specific focus on the repurposing potential of anti-psychotic, anti-inflammatory and anti-viral drugs, anti-diabetic, antibacterial, and anti-fungal drugs.

## Introduction

Cancer is associated with a condition wherein there is abnormal proliferation of cells and lack of proper regulation of cell cycle and apoptosis. It is characterized by dispersion of cancer cells to different tissues and organs known as ‘metastasis’, and invasion to other regions by inhibition of cell to cell contact and cell to matrix contact. It is through uncontrollable metastasis that increases the spread of the disease, consequently reducing the treatment effectiveness. With due course of time, cancer cells develops resistance to chemotherapeutical drugs through a multitude of mechanisms which results in cancer progression and treatment failure along with poor vascularization, intratumoral high interstitial fluid pressure, hypoxia, and phenotypic resistance due to toxicity induced by drugs [1, 2]. Accordingly, there is a requirement to identify drug alternatives to counteract the increase in drug resistance and to improve the control over the spread of the disease.

Drug repurposing, also known as drug reprofiling or drug repositioning, is one of the most interesting methods used to identify new therapeutic applications for existing clinically approved drugs. This strategy aims to reduce the cost, labour and research time considerably. As compared to the extremely time consuming, expensive and risk-based traditional drug discovery and development process, drug repurposing has proven to be efficient, cost effective and less in toxicity [3]. Making use of drugs that have already been clinically tested for humans gives a better understanding of the pharmacokinetics, pharmacodynamics, dose, metabolic profiles, molecular functional mechanism of the drugs, as well as off-target interactions. Drug repositioning frequently uses computational, experimental, and combination approaches for identifying a suitable drug to be repurposed. The repurposed drugs have constituted around 30% of all drugs approved by US FDA (United States Food and Drug Administration) and vaccines, accounting 25% of total annual revenue of the pharmaceutical industries [3, 4].

Some of the most prominent examples of drug repurposing include sildenafil, aspirin, minoxidil, valproic acid, etc. Sildenafil commonly known as Viagra was originally developed as anti-hypertensive for treating hypertension and angina pectoris. However, during the phase II clinical trials, it was revealed that sildenafil caused penile erections. Hence, it was repurposed for treating erectile dysfunction [5, 6]. In recent years, drug repurposing in oncology has been gaining attention due to the increasing reports of resistance to well-established drugs in various cancers and also to develop better treatment regimen with less or no adverse side effects in cancer patients [7, 8].

The expanding digitization of data, which can be acquired from a variety of sources spanning through pharmaceutical sciences to cheminformatics-directed databases, has changed medical discovery. Currently, high performance computing platforms and openly accessible resources like biological, physiological and clinical data may be combined to create a complete map of signalling pathways and pharmacological modes of action in regards to drug candidates [9]. Rapid innovations in computational data mining have enhanced assessments of ‘big data’ models, and the finding of novel applications for already approved drugs has been advanced. Computational drug repurposing is categorized into two types: exploring different applications for a current drug and identifying potent drugs for an illness, with the common technique of assessing similarities between drugs and/or illnesses. The following two technology developments have enabled the development and widespread usage of computational drug repurposing. The first development is the generation and accumulation of high-throughput data from multiple sources, such as genomics and proteomics. As a result, not just knowledge on disease phenotypes and pharmaceutical assessments, but also whole route maps, are now available. The second is that improvements in computational and data sciences have enabled the development of repurposing algorithms, as well as retrospective analysis and database management for experimental results [10].

With the rise of drug-related statistics and open data programs, a range of novel repurposing tactics and methodologies for combining data from multiple sources, including as pharmacological, genetic, chemical, or clinical data, has evolved like knowledge-based repurposing, target-based drug-repurposing, pathway-based drug-repurposing, gene expression profiles, network analysis, and protein-ligand docking studies.

Knowledge-based repurposing makes use of drug-related data such as drug targets, chemical structures, pathways, harmful effects, and so on. Models are created to forecast undiscovered targets, biomarkers, or disease pathways. Considering proteins or biomarkers of relevance, target-based drug repurposing entails high-throughput screening (HTS) of drug compounds, accompanied by in silico screening of drug candidates from drug databases, such as ligand-based screening or docking [11]. Gene expression profiling is the analysis of the sequence of genes expressed at the transcriptional level under various conditions or in a given cell to provide a wider view of cellular activity. To determine gene expression, various approaches are utilized. DNA microarrays and sequencing technology are examples of this. The former monitors the function of particular genes of interest, whereas the latter determines the function of all active genes in a cell [12]. WGCNA (weighted gene co-expression network analysis) is a bioinformatics tool used to investigate the links between distinct gene sets (modules) or between gene sets and clinical characteristics. Due to their capacity to incorporate information from multiple sources, network-based techniques are commonly employed in drug repurposing. For small-molecule ligands to macromolecular targets, virtual docking can be utilised to predict binding conformations and free energies of binding [13]. Docking is frequently applied in this research of biomolecular activities and interactions, as well as in the development of structure-based drugs. The technologies are rapid enough to enable high-throughput screening of tens of thousands of molecules in ligand databases. Such a protocol describes the docking and computer-aided drug screening methods provided by the AutoDock toolset of programs, such as basic docking of a therapeutic agent with an anti-cancer target, computational screening of this target with a small ligand database, docking with specific receptor flexibility, active site estimation, and docking with overt hydration [14].

For computational drug repurposing studies, various types of big data are openly available. Multimodal repurposing methodologies for diverse data can aid in the discovery of new applications for existing pharmaceuticals. Thus, drug repurposing has the potential to speed up drug discovery for cancer and infectious disorders. The present review focuses on therapeutic repurposing of anti-psychotic, anti-inflammatory, anti-viral, anti-diabetic, anti-bacterial, and anti-viral drugs as an alternative pharmacological strategy against human cancers.

### Anti-psychotic drugs

The increasing severity of drug resistance in cancer and the high cost of developing new drugs has resulted in a surge in research for finding alternative, economical and low-toxicity associated anti-cancer drugs [15]. In addition to neuroleptic effects, anti-psychotic drugs have also been reported to perform efficiently against different types of cancers.

Several studies have demonstrated that patients taking anti-psychotic drugs for psychiatric diseases such as Schizophrenia have decreased incidences of colon, rectal and prostate cancers [16–18], suggesting that anti-psychotic drugs do have an anti-cancer potential [19, 20]. Aripiprazole, a drug given to schizophrenic patients, slows down the proliferation of cells and tumor growth in colon, glioma, and gastric cancer [21]. Sertindole, a drug used in Schizophrenia, is also a promising candidate drug in treating breast and gastric cancers [22, 23].

At the molecular level, anti-psychotic drugs have been observed to influence multiple pathways that lead to the death of tumor cells [15]. Valproic acid, a common neuroleptic agent used for curing epilepsy, bipolar disorder, and migraines, has been observed to utilize epigenetic mechanisms such as inhibition of histone deacetylases that further results in decreased proliferation of tumor cells, triggers cell differentiation, inhibits angiogenesis ultimately resulting in cell death [24–26]. Phenothiazine used in the treating schizophrenia acts as an antagonist of dopamine receptor. Phenothiazines have been stated to induce differentiation of tumor stem cells, inhibit DNA polymerase in mitochondria, and reduce proliferation of tumor cells [27]. Olanzapine is used in the treatment of Bipolar disorder, Schizophrenia and Tourette syndrome. It works as an antagonist of the 5-hydroxytryptamine (HT) 7, D2, D3, and D4 receptor. It kills tumor cells by disrupting cholesterol homeostasis [28]. Selective serotonin reuptake inhibitors (SSRI) has been shown to decrease the proliferation thereby inducing tumor cell death [29]. Tricyclic anti-depressants block noradrenaline and serotonin transporters which results in the increase of neurotransmission. They inhibit proliferation of cells and induce apoptosis in different types of cancers [30]. Monoamine oxidase inhibitors (MAOIs) selegiline, phenelzine, and tranylcypromine prevent monoamine neurotransmitters from breaking down which are responsible for inhibiting BHC110/LSD (lysine specific demethylase 1, also known as BHC110). BHC110/LSD1 has the ability of causing histone demethylation which leads to cancer progression, metastasis, and therapy resistance [31, 32]. Therefore, by inhibiting BHC110/LSD1, tumor progression can be inhibited.

Several anti-psychotic drugs such as brexpiprazole, aripiprazole, and sertindole have demonstrated anti-cancer potential in both in silico as well as in vitro studies. Brexpiprazole is used in the treatment of schizophrenia. It is known to modulate the activity of serotonin and dopamine. It has both blocking as well as the stimulating activity as it acts as a partial agonist of 5-HT1A and D2 receptors besides acting as an antagonist of norepinephrine alpha 1B/2C and 5-HT2A receptors [33, 34].

**Fig. 1:**
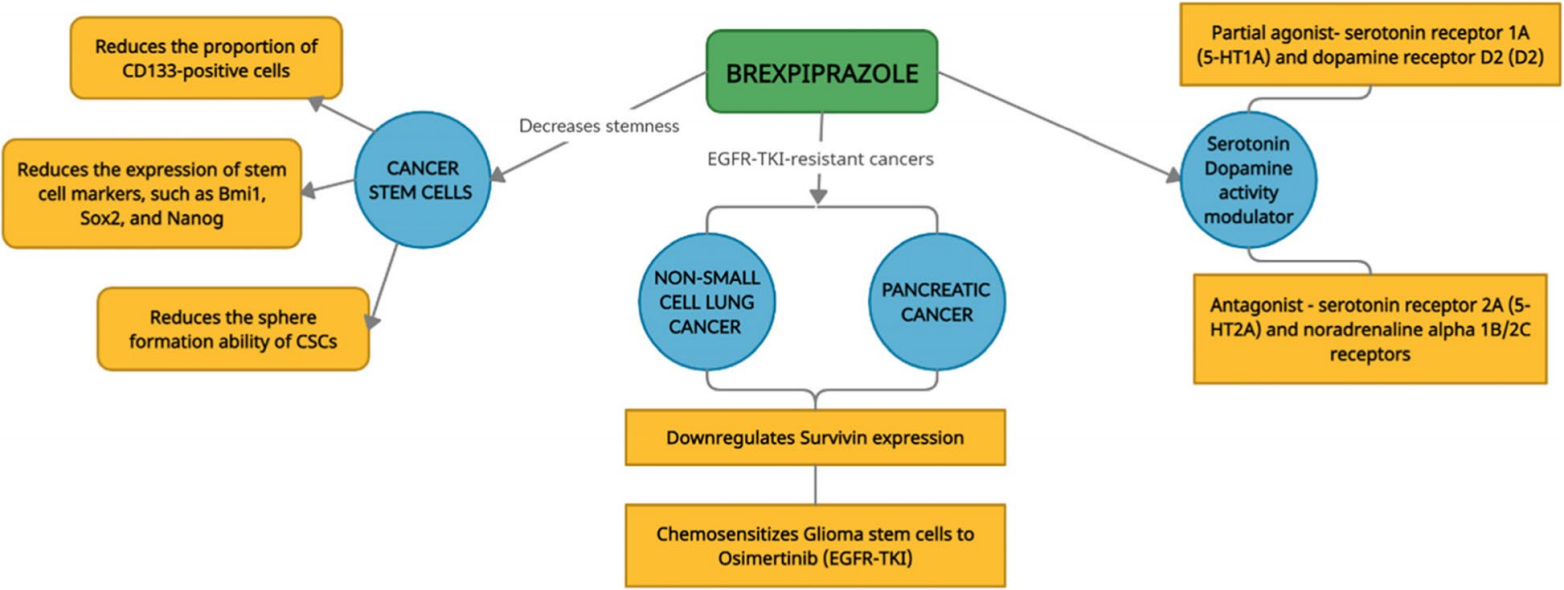
Mechanism of action of Brexpiprazole in cancer. Brexpiprazole modulates the activity of serotonin and dopamine; downregulates expression of CD133+ cells, Bmi1, Nanog, and Sox2; and reduces sphere formation ability of CSCs

Brexpiprazole shares chemical and pharmacological similarity with aripiprazole, which was the first D2 partial agonist available in the market. Brexpiprazole has low intrinsic activity at D2 and D3 dopaminergic receptors, thus the toxicity profile of brexpiprazole is better as compared to aripiprazole [35, 36]. Glioblastoma frequently develops resistance against EGFR-tyrosine kinase inhibitors (EGFR-TKIs). Brexpiprazole acts as chemosensitizer in pancreatic cancer and non-small cell lung cancer by downregulating expression of survivin which is an anti-apoptotic protein and thus it chemosensitizes the glioblastoma stem cells to osimertinib which is an EGFR-TKI. Brexpiprazole also reduces the proportion of CD133+ cells, decreases the expression of stem cell markers Bmi1, Nanog, and Sox2, and it negatively impacts the sphere formation ability in cancer stem cells (CSCs) which ultimately affects the self-renewal ability of CSCs [37, 38]. The potential mechanism of action of brexpiprazole in multiple cancers is summarized in Fig. 1.

Aripiprazole is a drug prescribed to patients suffering from bipolar disorder and schizophrenia. It is a partial agonist of Dopamine D2 receptor. It slows down the proliferation of cells and tumor growth in colon, glioma and gastric cancer [39]. A recent study [40] has shown that when the PANC-1, PSN-1, and A549 human cancer cell lines were treated with aripiprazole, it was able to kill these cells at a concentration which was non-toxic to the normal cells. Aripiprazole induces apoptosis in both cancer cells as well as cancer stem cells. It also differentiates cancer stem cells into non-cancer stem cells. It downregulates the expression of survivin which is an anti-apoptotic protein and therefore sensitizes the cancer cells to chemotherapeutic agents [35].

Sertindole is also a promising candidate drug for treating breast and gastric cancers [41]. It bears a high affinity for dopamine D2, serotonin 5-HT2A and 5-HT2C, and α1-adrenergic receptors. Studies [42] have demonstrated that sertindole induces production of ROS which in turn cause autophagy and associated cell death in neuroblastoma cells. Sertindole has also shown evidences of preventing breast to brain metastasis. This is attributed to the fact that all the anti-psychotic drugs can cross the blood brain barrier [42].

The prospect of studying new clinical indications for the anti-psychotic drugs is alluring as they have shown pronounced effects as potential anti-cancer agents. Rapid translation can occur for these drugs as they have already proven to be safe in humans.

### Anti-inflammatory drugs

Anti-inflammatory drugs (more commonly known as non-steroidal anti-inflammatory drugs (NSAIDs)) are well known drugs used for reducing fever, inflammation, as well as pain-related conditions like chronic pain, acute headache, dysmenorrhea, and postoperative pain [43, 44]. It is one of the most commonly used class of drugs, consumed by approximately 30 million people across the globe. There are various types of NSAIDs in the market and they are segregated from each other on the basis of structure and associated risks.

Almost all NSAIDs are completely absorbed, i.e., they are bound to the serum proteins (albumin, globulin) tightly and have small volume of distribution [45]. Besides showing an anti-inflammatory effect, the NSAIDs also possess analgesic, anti-pyretic, and anti-thrombotic properties. In the current COVID-19 pandemic scenario, NSAIDs have been routinely administered to patients as anti-pyrexic and for reducing muscle pain. Although prolonged or excessive usage of these agents has many adverse effects like suppression of memory and synaptic plasticity, gastrointestinal ulcers with bleeding, perforation or obstruction, renal dysfunction, cardiovascular disorders, as well as risk of death [46–48].

NSAIDs, when incorporated in a patient, works by inhibiting enzyme cyclooxygenase (COX), which synthesizes prostaglandins and thromboxane, thereby mitigating the probable symptoms. Prostaglandins are responsible for inflammation, which they regulate through vasodilation and platelet aggregation. They are synthesized by nucleated cells and hence are found very commonly within the body [45]. Thromboxane helps in activating platelets, promotes platelet aggregation, and acts as a vasoconstrictor after tissue injury and inflammation [49]. The COX enzyme has two isozymes, namely, COX-1 and COX-2, categorized according to their selectivity. COX-1 is non-selective and is constantly expressed in the system. It produces prostaglandins and thromboxane A2 which are necessary for maintaining lining of gastrointestinal mucosa, aggregating platelets and other physiological functions. COX-2 is selective and is inducible in the presence of prostaglandins mediated pain and inflammation [48, 50, 51].

Interestingly, over the years, there are a number of reports and articles [52] linking chronic inflammation with possible cancer risk. The promotion of cancer progression is stated to be initiated by exposure of chemical irritants like phorbol esters, certain hormones, and factors at the wounding site. The macrophages and leukocytes present at the inflammation site, generates reactive oxygen and nitrogen species along with mutagenic agents that could damage tissue and change base pairings in DNA [52]. There are various inflammatory mediators that promotes tumor development, namely cytokines, chemokines, growth factors, and proteolytic enzymes. These mediators enables cancer cells to survive by promoting their proliferation and accumulating mutations. The inter-relationship between inflammation with gut microbiota, internal and external environmental factors, and genetics of the host is responsible for the development of colorectal cancer (CRC) [53]. The cancer cells are characterized by the production of cytokines and chemokines and increased expression of certain immune cells and fibroblasts. Also, statistics suggests that around 20% of all cancer types arise from chronic inflammatory disease [54].

The effect of an NSAID, named mesalazine (also known as mesalamine or chemically written as 5-aminosalicylic acid (5-ASA)), on CRC and ulcerative colitis (UC) has been reported. As per a rodent model study, it inhibits growth of tumor and lowers the number of aberrant crypt foci [55]. The recurrence of familial adenomatous polyposis (FAP) was reduced by the use of sulindac, while ibuprofen and piroxicam lowered the probable risk of breast and CRC occurrence [56]. Recent in vivo data suggests that diclofenac successfully inhibited the progression of pancreatic tumors in mice. Analysis of the excised tumor tissue revealed that the treatment with diclofenac caused increase in apoptosis and decrease in angiogenesis. Also, a combination of diclofenac with sorafenib (a kinase inhibitor) was tested on melanoma cells, and it showed efficacy against all cancer cells [57].

Additionally, celecoxib, a selective COX-2 inhibitor suppressed breast cancer cells growth and reduced tumor development in in vivo rat models. It inhibited cell growth by inhibiting and decreasing the expression of aromatase and estrogen receptor α. The action of the drug was studied on two human breast cell lines, one being of very invasive nature MDA-MB-231 and the other being moderately invasive MDA-MB-468. It was seen that the suppression of growth was dependent on COX-2 expression levels and the invasiveness of the tumor cells [58]. All these studies indicate towards the existence of a concordance between the use of NSAIDs and reduced cancer progression.

**Fig. 2:**
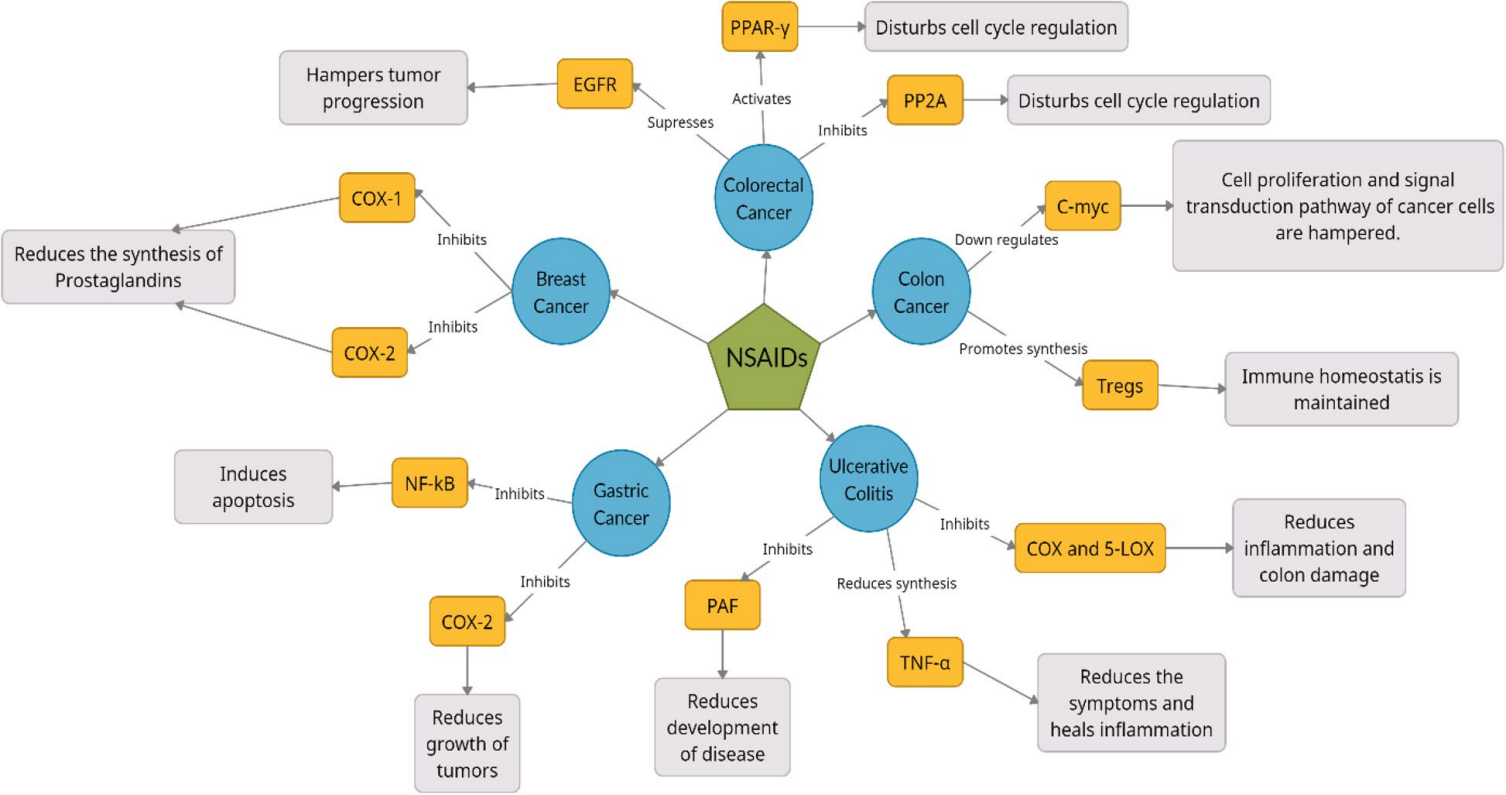
Schematic representation of various action of NSAIDs through different targets and its consequences in various conditions and cancer types. NSAIDS have been known to inhibit NF-KB, COX-1, COX-2, 5 LOX, PAF, and PP2A respectively, and supress EGFR in different cancer types

NSAIDS, specifically mesalazine have been reported to have anti-cancer potential in different cancer as represented in Fig. 2. For examples in colorectal cancer, PPARs are the transcription factors that are activated by the drug to inhibit Wnt/Beta-catenin pathway and down-regulate COX-2 [59, 60]. The function of PP2A (a serine/threonine phosphatase) is interfered by the drug via mitosis and enabling apoptosis [61, 62]. Overexpression of EGFR is suppressed to inhibit the recurrence of cancer [63, 64]. Similarly in colon cancer, C-Myc is a transcription factor, the blockage of which is required for hampering the cell proliferation and signal transduction pathway across the cancer cell [65, 66]. The drug promotes the accumulation of T_regs_ (regulatory T cells) through TGF-β by activating a receptor called Ahr (aryl hydrocarbon receptor) to suppress excess of immune response [67–69]. In ulcerative colitis**, t**he function of TNF-α is changed (reversed), making it harmful for cancer cells [70]. COX, 5-LOX pathways and PAF are inhibited as their further metabolism could turn normal cells to cancer cells [71]. Gastric cancer and breast cancer- COX inhibition is seen in both cancer types, apart from UC. NF-κB acts as an important target, as most of the molecules formed by transcription of genes lead to gastric cancer [72, 73].

NSAIDs, specifically selective COX-2 inhibitors shows promising anti-cancer activity as proven from various experiments and clinical studies. However, its dose, regimen of treatment, risks, and benefits need to be clarified.

### Anti-viral drugs

Anti-viral drugs are mostly formulated with the purpose of targeting different human infecting viruses like HIV, herpes, hepatitis, and influenza. Instead of destroying their target viruses that have infected the cells of the body, these drugs act by repressing the duplication and further development of these viruses [74, 75]. Anti-virals have also been repurposed to treat various infections, diseases, etc. and there are many prominent examples of the same.

The flu-treatment medicine favipiravir, and the hepatitis C therapy drug sofosbuvir, both have significant promise for remodeling against Ebola and Zika infections [75]. Cidofovir is a drug that is used to treat cytomegalovirus (CMV) as well as other DNA viral infections. It has been utilized to treat JC virus-related PML (progressive multifocal leukoencephalopathy), HSV sickness that is resistant to acyclovir, BK virus-related hemorrhagic cystitis, and other double-stranded DNA virus diseases [75]. Similarly, many anti-viral medications that are currently accessible have been repurposed to treat a variety of viruses and disorders [76, 77]. With the onset of COVID-19, many anti-viral drugs like ritonavir and remdesivir have also come in handy for treating and reducing transmission of SARS-CoV-2 virus [78].

Anti-viral agents have been recently studied for their anti-cancer properties. Among them, the anti-HIV medications target a variety of mechanisms in the HIV life cycle, providing a broad pool of potential chemotherapeutic agents [79]. A study revealed that acyclovir as an anti-viral specialist has potential benefit when used as an adjuvant for treating breast cancer [74]. Breast cancer cells have been observed to grow and proliferate at a slower pace when treated with this anti-viral medication, which regulates the production of cytokines, associated with apoptosis, that is Caspase-3. It interdicts and prohibits the cancer cell proliferation and formation of colony and cell invasion, and shows no effect on secretion of tumor separation genes [74].

By reverse transcriptase, azido thymidine was the first medicine licensed for the treatment of HIV infection. This drug also tends to show anti-cancer effects against a number of cancer types like leukemia, lymphoma, Kaposi sarcoma, and pancreatic cancer [79]. A rise in telomerase activity, as well as inhibition, is observed in cancer cells, which leads to apoptosis [79, 80]. Brivudine, an anti-viral drug used for herpes simplex virus, too showed anti-cancer properties by suppressing the chemoresistance [75]. Brivudine has been shown to successfully limit the proliferation of bortezomib-resistant MM cells when used in combination with bortezomib [81]. A study to evaluate effective and safe RP101 (Brivudine) is when used in conjunction with Gemcitabine to treat pancreatic cancer, is in phase II now [82]. Nelfinavir, a protease inhibitor, has been shown to be efficacious against HIV-1 and HIV-2 infections. It has shown potent anti-cancer effects, by inhibiting Akt-signaling and inducing endoplasmic reticulum (ER) stress as prime mode of action [83]. In NSCLCs, ovarian cancers, liposarcomas, as well as breast cancers, nelfinavir induces ER stress and the consequent unfolded protein response (UPR) [84]. In vitro and in vivo, nelfinavir/COX-2 suppression may enhance the lethal effects of medicines that suppress autophagy in triple negative breast cancer [85]. A phase II clinical trial study of nelfinavir in patients with locally advanced head and neck squamous malignancy is now underway [86]. Treatment of patients diagnosed with AML (acute myeloid leukemia) with the anti-viral ribavirin has demonstrated effectiveness. Ribavirin also lowered the levels of oncogenes (cyclin D1 and NBS1) by interfering with eIF4E for mRNA binding and altering eIF4E's structure [87]. Ribavirin also has a role in immune modulation and anti-proliferative action by targeting inosine monophosphate dehydrogenase [88, 89]. A recent study revealed the capability of ribavirin as an anti-cancer specialist in five human Nasopharyngeal Carcinoma (NPC) cell lines. Utilizing cell development measures and other assays, it was reported that in vitro, ribavirin diminishes NPC cell multiplication, movement, and intrusion and advances cell-cycle arrest and apoptosis. Ribavirin diminished cancer growth in numerous NPC xenograft models in vivo, as monotherapy. In vitro and in vivo, ribavirin improved the effects of radiation, which is a key component of NPC treatment. This study suggests that clinical trials of ribavirin for its anti-NPC properties be conducted [88]. The efficacy of combining zidovudine, ganciclovir, and interleukin-2 in the treatment of people with primary central nervous system lymphoma which is related to AIDs is now being studied in a phase II trial [89]. Lamivudine, another anti-viral medication that may be able to prevent cancers from developing drug resistance. This drug’s efficacy as a therapeutic agent for radio sensitization of esophageal squamous cell carcinoma has been demonstrated in one study [90]. Combining lamivudine with standard chemotherapeutic drugs may contribute, to decrease tumor cell development and spread to other regions of the body and a phase 2 trial of the same is ongoing as well [91].

**Fig. 3:**
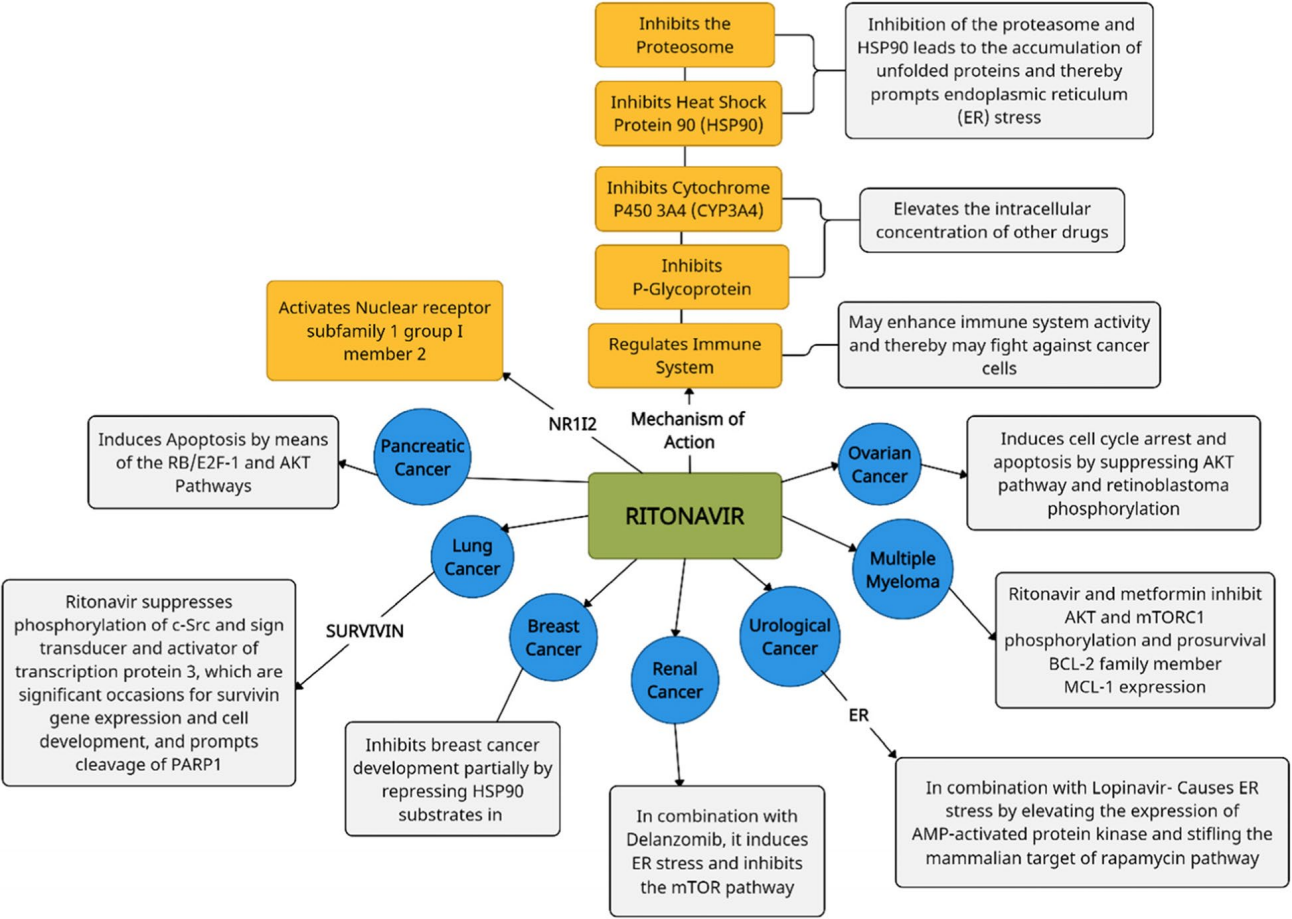
Pictorial representation of diverge mechanisms of action of ritonavir in different cancers. Ritonavir is thought to apply its anti-cancer action by hindering numerous signaling pathways, including the AKT and nuclear factor-kappa B pathways; it also inhibits survivin, HSP90 substrates and induces ER stress and apoptosis by blocking Akt pathways in various cancers

Ritonavir is one of the widely used anti-viral drugs for HIV treatment [92]. Ritonavir has been shown to impede cell cycle progression, induce apoptosis, and affect metabolism in a multitude of ways. Ritonavir is thought to apply its anti-cancer action by hindering numerous signaling pathways, which includes the AKT and nuclear factor-kappa B pathways [93]. It activates the NR1I2 gene and represses cytochrome P450, P-glycoprotein, the proteasome, and HSP90 [94]. In clinical settings, it has also been used as a stimulant to activate additional anti-viral drugs. Various studies have shown that ritonavir can be repurposed for treating cancer. The action and targets of ritonavir when administered to treat various cancers have been explained schematically in Fig. 3. Ritonavir-induced phosphorylation (inactivation) of AKT could point to a possible therapy for pancreatic cancer [95]. Ritonavir inhibits lung cancer cells partially by suppressing survivin [96]. In breast cancer, ritonavir affects Akt-regulated cell proliferation by inhibition of HSP90 substrates [93]. The first phase of a trial evaluating the impact of Ritonavir on biomarkers in women receiving surgery for early stage breast cancer detection has now been completed [97]. The blend of ritonavir and delanzomib synergistically incited endoplasmic reticulum stress and repressed the mammalian objective of rapamycin (mTOR) pathway in renal cancer cells [98]. Lopinavir and ritonavir, when taken together, act against urological cancer cells by inciting ER stress synergistically [99]. Ritonavir and metformin successfully focus on multiple myeloma cell metabolism in order to evoke cytotoxicity in multiple myeloma [100]. Ritonavir has been shown to block AKT signaling which in turn leads to induction of apoptosis and hence, restrains the relocation and attack in ovarian malignant growth cells [101].

In ovarian, pancreatic, as well as breast cancer cells, ritonavir has been shown to decrease cancer cell growth and division and to accelerate apoptosis [102]. Administration of ritonavir lowers the levels of the glucose transporter GLUT4. Ritonavir medication may reduce tumor cell viability since many tumor cells depend on enhanced glucose metabolism [97]. Ritonavir also activates the NR1I2 gene (nuclear receptor subfamily 1 group I member 2), the transcriptional modulator of the cytochrome P4503A4 (CYP3A4). Ritonavir acts by inhibiting P-glycoprotein and CYP3A4 as this causes certain unfolded proteins to accumulate and thereby cause stress to the endoplasmic reticulum [94]. Ritonavir also inhibits the proteasome and heat shock protein 90 (HSP90), which leads to the increase of the concentration of other drugs intracellularly. Ritonavir has been proven to modulate the immune system, which might assist it in eliminating cancerous cells [94].

Despite the overwhelming amount of evidence establishing the advantageous impacts of anti-viral drugs in cancer therapy, the remedial benefit of their usage in disease treatment stays a hazy situation because of the absence of investigations of the biochemical systems. Anti-viral medications such as acyclovir, azido thymidine, and ribavirin have been shown to have a restrictive effect on the growth and potential to enhance apoptosis in various cancers. More large-scale studies are important to affirm the viability of these anti-viral treatments. Ritonavir is also another promising therapeutic agent to treat cancer.

### Anti-diabetic drugs

Diabetes is one of the ubiquitous clinical conditions causing to around 400 million people worldwide with a net expenditure of around 550 million dollars, determined by the International Diabetes Federation (IDF). It is a condition wherein the glucose in the blood is elevated to abnormal levels thereby exposing the patients to insulin resistance as well as cardiovascular and neurological disorders [103, 104]. The associated imbalance in the metabolism related to this disease exposes the patients to extremely greater risk vascular complications, like cardiovascular disease, resulting in frequent hospitalization, if not properly taken care [104]. It came as a surprise to the researchers that certain anti-diabetic drugs showed anti-cancer activity to an extent.

Metformin is an orally taken medication and used as first line of treatment for type 2 diabetes mellitus. It reduces the process of gluconeogenesis in the liver and enhances the insulin sensitivity. The anti-neoplastic activity is observed in various cancer types, such as, prostrate, breast, lung, endometrial and pancreas. On a general basis, there are two modes of action for Metformin to supress tumorigenesis. First, through lowering insulin, leading to supressed tumor progression and second, by directly acting on the cancer cells, specifically in the electron transport chain of the mitochondria, thereby reducing energy consumption. In breast cancer, it activates AMPK signalling pathway, which in turn inhibits mTOR pathway, inducing anti-cancerous activity [105–107].

Thiazolidinediones (TZDs) has identified to be a potent lead in treating breast and prostate cancer through various preclinical and clinical studies. It consists of three important compounds, namely, troglitazone, rosiglitazone, and pioglitazone [104, 106, 107]. The group induces anti-cancer activity by two ways: PPAR γ (peroxisome-proliferator-activated receptor gamma) dependent and PPAR γ independent mode. TZDs activates the PPAR γ receptor by bringing about a conformational change, which later forms a heterodimer with the retinoid X-receptor. The complex activates the transcription of target genes resulting in decreased cell proliferation and increased differentiation and apoptosis. TZDs acts independent of PPAR γ by relying on expression of PTEN/AMPK, AKT (protein kinase B)/mTOR (mechanistic target of rapamycin) and degradation of cyclins, D1 and D3 [104, 106, 107].

### Anti-bacterial drugs

Anti-bacterial drugs are the compounds that are used for treating infectious diseases caused by gram-negative, gram-positive bacteria, rickettsia, mycoplasma, chlamydia, and spirochaetes [108]. Usually, a bacterial infection leads to the secretion of protein toxins, which are sometimes used to form an environment favourable for carcinogenesis. It can be caused due to certain reasons like, genomic instability, resistance to cell death or proliferative signalling [109].

Clarithromycin, an FDA-approved anti-biotic drug, marketed with the name ‘Biaxin’, underwent clinical trial in 1997 for the first time in order to show its efficacy in curing lung cancer. It was seen that the anti-tumor activity of the drug was much more beneficial when used in combination with another drug. Doxorubicin, one of the subclass of anti-biotics was found beneficial for treating breast cancer. It worked by causing breaks in the DNA by intercalation and thereby inhibiting DNA replication [110, 111].

Doxycycline is a broad-spectrum anti-biotic marketed with various brand names such as ‘Acticlate’, ‘Dorxy’, and/or ‘Atridox’. It hampers tumor cells by blocking iNOS (nitric oxide synthase) necessary for the development of tumor, progression, growth, and angiogenesis. It inhibits growth of colon cancer cells by causing G0/G1 arrest and inhibiting matrix metalloproteinase, when used in combination with COX-2 inhibitor. In case of breast cancer, doxycycline inhibits the stem cell phenotype of cancer cells as well as the process of mitochondrial biogenesis [110, 111].

### Anti-fungal drugs

Fungal infections have constituted around 2 million deaths worldwide annually. They are usually diagnosed in people with compromised immune system and who have undergone organ transplantation. Some of the conditions like Aspergillosis, Candidiasis, and Mucormycosis have reported to have touched 300,000, 750,000, and 10,000 cases, respectively along with high mortality rate [112]. Anti-fungal drugs, too, are included in long list of repurposed drugs.

Itraconazole is known to inhibit AKT/mTOR signalling pathway in human umbilical vein endothelial cells (HUVECs), endometrial carcinoma (EC), melanoma cells, and glioblastoma. It reverses chemoresistance induced by P-glycoprotein, regulating the signal transduction pathways of Hedgehog, inhibiting angiogenesis and lymph angiogenesis of cancer cells [113, 114].

The off label use of ketoconazole, a broad spectrum anti-fungal agent, showed considerable anti-cancer activity for prostate, melanoma, breast, and hepatocellular carcinoma. In prostate cancer cells, it effectively inhibits the exosome biogenesis, with overall better tolerance and lesser side effects [115].

Mebendazole, another FDA-approved anti-fungal agent, showed anti-cancerous activity for non-small cell lung cancer (NSCLC) through tubulin depolymerization-induced cell cycle arrest. Additionally, it radio-sensitizes the breast cancer cells and reduces the proportion of stem cells through hedgehog pathway in order to respond to DNA damage [113].

## Conclusion

The various classes of drugs examined in this review have been exhibited to target the key genes in cancer pathogenesis. Anti-psychotic drugs used to treating neurological disorders proves to be a promising class of drugs for repurposing in cancer. These drugs inhibit proliferation of cancer cells and induce apoptosis in pre-clinical studies, with certain further investigations required to elucidate the exact mechanisms and targets of these psychiatric medications. The repurposing nature of these therapies will provide a quick translational time frame due to the pre-known mode of action and toxicology. We expect that many of these anti-psychotic agents will enter the market in times to come and provide treatments to extend the survival of many patients who suffer cancer with limited therapeutic options. Major work and research are undergoing in order to study the drug repurposing on cancer and many diseases possible, with its significance and consequences. It demonstrates that inflammation is one of the causes of cancer, countering which anti-inflammatory drugs decreased its occurrence. Additionally, the use of anti-inflammatory drugs combined with other conventional anti-cancer agents is getting momentum and is expected to add to new therapeutic approaches to treat cancer in next few decades. From the aforementioned results from various studies, we can state that anti-viral drugs can possibly be used in cancer therapy. Drugs like acyclovir, ribavirin, lamivudine etc. have shown anti-cancer properties. Thus, further investigation for more viable and advantageous anti-viral regimens warrants more consideration. Thus, it can be proposed that anti-cancer medications that can be administered in combination with an anti-psychotic, anti-inflammatory, anti-viral drugs, anti-diabetic, anti-bacterial, and anti-fungal work independently, irrespective of the type of cancer and further exploration on this could yield favorable results.

## Data Availability

The datasets used and/or analyzed during the current study are available from the corresponding author on reasonable request.

## Abbreviations

5-HT: 5-hydroxytryptamine
SSRI: Selective serotonin reuptake inhibitors
MAOIs: Monoamine oxidase inhibitors
LSD: Lysine specific demethylase
EGFR-TKIs: EGFR-tyrosine kinase inhibitors
CSCs: Cancer stem cells
NSAIDs: Non-steroidal anti-inflammatory drugs
COX: Cyclooxygenase
CRC: Colorectal cancer
5-ASA: 5-aminosalicylic acid
UC: Ulcerative colitis
FAP: Familial adenomatous polyposis
T_regs_: Regulatory T Cells
Ahr: Aryl hydrocarbon receptor
CMV: Cytomegalovirus
PML: Progressive multifocal leukoencephalopathy
ER: Endoplasmic reticulum
UPR: Unfolded protein response
AML: Acute myeloid leukemia
NPC: Nasopharyngeal carcinoma
mTOR: Mammalian objective of rapamycin
CYP3A4: Cytochrome P4503A4
HSP90: Heat shock protein 90
